# From Physiology to Practice: Validation of Eccentric Velocity Monitoring Using an Optoelectronic System

**DOI:** 10.3390/s26061797

**Published:** 2026-03-12

**Authors:** Fernando Martin-Rivera, Darío Rodrigo-Mallorca, Jose Vidal-Vidal, Luis M. Franco-Grau, Angel Saez-Berlanga, Iván Chulvi-Medrano

**Affiliations:** 1 Department of Physical Education and Sports, University of Valencia, 46010 Valencia, Spain; fernando.martin-rivera@uv.es (F.M.-R.); dariorodrigom@gmail.com (D.R.-M.); luis.m.franco@uv.es (L.M.F.-G.); ivan.chulvi@uv.es (I.C.-M.); 2 Department of Sports Sciences, University of Miguel Hernández, 03202 Elche, Spain; jose.vidalv@umh.es

**Keywords:** eccentric phase monitoring, velocity-based training, linear position transducer, back squat, measurement validity, reliability

## Abstract

**Highlights:**

**What are the main findings?**

- PowerTrack^TM^ showed very high validity for eccentric phase velocity during the Smith machine back squat when compared with the criterion system (MuscleLab^TM^), particularly for eccentric peak velocity.
- Inter-session reliability was excellent for eccentric velocity outcomes, supporting the device’s practical use for eccentric velocity-based training in applied settings.

**What are the implications of the main findings?**

- Eccentric–phase velocity during the Smith machine squat can be monitored with high validity and reliability using an PowerTrack^TM^ optoelectronic sensor.
- Eccentric peak velocity (Vmax) appears to be a more robust metric than eccentric mean velocity (MV) for monitoring eccentric actions in multi–joint resistance exercises.

**Abstract:**

Background: Accurate monitoring of eccentric phase velocity is needed to support velocity-based training (VBT), yet field-valid tools for multi-joint exercises are scarce. This study evaluated the concurrent validity and reliability of an optoelectronic device to quantify eccentric phase velocity during the Smith machine back squat. Methods: A total of 20 resistance-trained men completed two sessions and performed three repetitions at three submaximal loads (20, 50, and 70 kg). Eccentric mean velocity and peak velocity (Vmax) were recorded simultaneously using PowerTrack^TM^ and a criterion system (MuscleLab^TM^). Validity was assessed using ordinary least products regression, Lin’s concordance correlation coefficient (CCC), and Bland–Altam analysis. Reliability was examined via intraclass correlation coefficients (ICC), standard error of measurement (SEM), coefficient of variation, and minimum detectable change. Results: Agreement between devices was very high for Vmax (slope ≈ 1.00; CCC = 0.95), with a small constant bias. Eccentric mean velocity showed proportional bias under higher velocity conditions, whereas overall concordance remained high (CCC = 0.95). Inter-session reliability was excellent (ICC = 0.87–0.96), with low SEM values for eccentric velocity metrics. Conclusions: PowerTrack^TM^ can be a valid and reliable tool for monitoring eccentric phase velocity during the Smith machine back squat, with Vmax representing the most robust metric for applied eccentric VBT.

## 1. Introduction

Resistance training constitutes a fundamental component for enhancing athletic performance and preventing conditions associated with physical function decline [1]. Among the phases of the muscle activation cycle, eccentric action (EP)—defined by the active lengthening of the muscle under load—exhibits unique physiological properties that augment force production, neuromuscular efficiency, and the mechanical stimulation of muscle and tendon tissue [2].

Historically, eccentric strength protocols were developed in laboratory settings using isokinetic devices that controlled angular velocity in single-joint movements [3,4]. These studies established the foundations of the hypertrophic and neuromechanical potential of eccentric stimuli, demonstrating their superiority, in certain cases, over concentric or traditional methods. In a recent review, it was concluded that eccentric exercise has a sarcomerogenic potential that may be one of the most influential mechanisms in promoting muscle optimization, rehabilitation processes, and injury prevention [5].

Traditionally, eccentric exercise has been applied using supramaximal loads (e.g., >120% 1RM) or by prolonging the eccentric phase in submaximal loads (e.g., for 6 s). In both situations, execution velocity is a key factor and should be properly monitored [6]. In the past two decades, the application of eccentric training has extended beyond elite sports settings [7], becoming integrated into health and rehabilitation programs [8], particularly among populations with specific needs such as older adults, individuals with sarcopenia, or patients with musculoskeletal disorders [2,9,10,11]. This expansion has created new methodological and technological demands regarding its prescription and quantification.

Despite advances in the use of velocity-based training (VBT) to monitor relative intensity and neuromuscular fatigue during the concentric phase [12,13,14], the eccentric component has remained less explored and rarely quantified objectively. Variations in the duration or intent of the negative phase have not always been translated into precise recordings of mechanical behavior, thereby limiting both the dosing and individualization of the stimulus [15,16].

The rise in emerging technologies—such as inertial sensors, optical cameras, and optoelectronic systems—has facilitated real-time performance monitoring in uncontrolled settings [6,17]. However, most available devices have been validated exclusively for concentric phases, lacking specific studies supporting their reliability and validity in assessing eccentric variables such as negative velocity, downward acceleration, or the eccentric-to-concentric ratio [17,18]. This gap represents a critical limitation for the effective implementation of VBT in field-based contexts; due to the importance of eccentric exercise, it is necessary to determine the execution velocity of this phase for appropriate prescription.

In response to this gap, the present study aimed to evaluate the validity and reliability of a next-generation optoelectronic device (PowerTrack^TM^) for monitoring movement velocity during the eccentric phase of the Smith machine squat. It was hypothesized that the device would demonstrate high agreement with a reference system (MuscleLab^TM^) and exhibit good inter-session consistency, thereby supporting its application in both sports and health settings for the accurate prescription of eccentric training.

## 2. Materials and Methods

### 2.1. Experimental Design and Rationale

The present investigation aimed to evaluate the reliability and concurrent validity of the PowerTrack^TM^ optoelectronic system for quantifying three velocity-related metrics—mean velocity (MV), and peak velocity (Vmax)—specifically during the eccentric phase of the back squat exercise on a Smith machine. After the recruitment period (2 weeks), participants performed two familiarization sessions and were assessed across two further sessions using an incremental loading protocol comprising three absolute loads (20, 50, and 70 kg). For each load, participants performed five repetitions, yielding a total of 300 repetitions. All repetitions, stratified by the measurement system (PowerTrack^TM^ and MuscleLab^TM^), velocity metric (MV, Vmax), and load, were included in the validity and reliability analysis.

**Figure 1:**
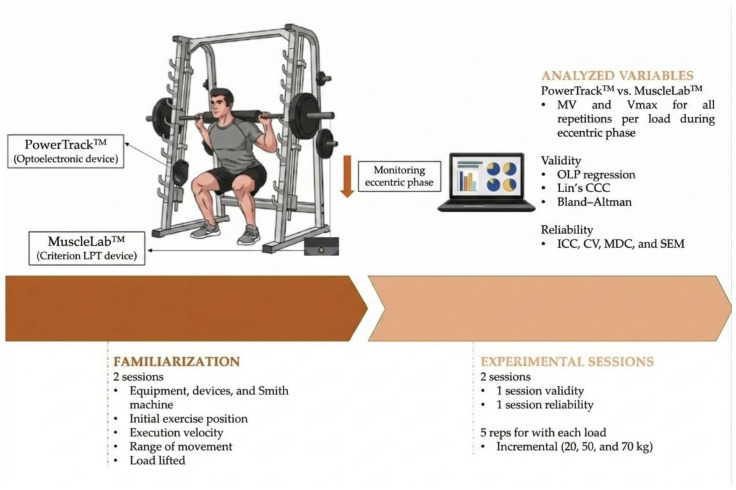
Study design. LPT, linear position transducer; MV, mean velocity; Vmax, maximum velocity; OLP, ordinary least products; CCC, Lin’s concordance correlation coefficient; ICC, intraclass correlation coefficient; CV, coefficient of variation; MDC, minimum detectable change; SEM, standard error of measurement; ↓, downward slope refers to the eccentric phase of the squat.

Crucially, selecting only the repetition with the best performance (e.g., the highest MV value obtained with MuscleLab^TM^) could have introduced bias, potentially inflating the reliability estimates for certain variables or systems. To mitigate this issue, all repetitions captured by the MuscleLab^TM^ system at each load were incorporated into the analysis. These same repetitions were subsequently used to assess the concurrent validity of PowerTrack^TM^ against the reference system. A schematic overview of the experimental procedures is provided in Figure 1.

### 2.2. Participants

A total of 20 recreationally active men [age = 36.80 (8.23) years; height = 1.72 (0.07) m; body mass = 74.36 (8.35) kg; body fat = 18.59 (7.46) %] volunteered to participate in this investigation. Recruitment was carried out at a local fitness facility, and all participants had at least one year of prior experience with the back squat exercise, ensuring familiarity with its technical execution during testing. Nevertheless, two standardized familiarization sessions were performed to guarantee full adaptation to the equipment, devices, and Smith machine; to standardize the starting position; to establish the prescribed movement velocity and range of motion; and to accustom participants to external loads used. Eligibility criteria required that participants had no musculoskeletal injuries, physical limitations, or health conditions that might affect performance outcomes. Before enrollment, all individuals received comprehensive information about the study procedures and provided written informed consent. The protocol adhered to the principles of the Declaration of Helsinki and received ethical approval from the Ethics Committee of the University of Valencia (reference: 2024-FIS-3642443).

### 2.3. Testing Procedures

The MV and Vmax of the EP were concurrently assessed using the MuscleLab^TM^ and PowerTrack^TM^ systems. For the criterion measure, a high-frequency linear position transducer (LPT) was secured to the right-side of the barbell via a tether, providing instantaneous vertical velocity signals at 200 Hz. This device was connected to a personal computer through a 14-bit analog-to-digital conversion interface and custom acquisition software (MuscleLab^TM^, Ergotech Innovation AS, Stanhelle, Norway), whose reliability and validity have been extensively documented in prior investigations [19].

In contrast, PowerTrack^TM^ (Tratech Technologies, Alicante, Spain) is a precision optoelectronic instrument designed to quantify resistance training parameters—including power, velocity, acceleration, and displacement—through a non-contact optical sensor. By avoiding cable mechanics, it minimizes errors associated with friction and trajectory constraints. The device can be magnetically affixed to plate-loaded machines, enabling stable and interference-free monitoring. Additionally, its proprietary software can provide individualized feedback and allow continuous data capture, thereby facilitating accurate tracking of EP performance for both experimental and applied training purposes. The validity and reliability of this system has been documented in a previous study [17].

From a technical standpoint, the PowerTrack^TM^ incorporates a programmable time-to-flight (ToF) laser-based ranging sensor that can permit modification of both acquisition frequency and detection range, thereby improving its suitable across diverse biomechanical applications. The system is capable of sampling at rates up to 50 Hz and can function in different operational modes: short distance (~1.3 m) with sub-millimetric precision (0.25 mm), medium distance (~3.0 m), and extended distance (~4.0 m) with optimized signal processing. This versatility can allow investigators to prioritize temporal resolution or spatial coverage according to the specific requirements of the EP task being assessed. Unlike the MuscleLab^TM^ unit, which records at a fixed 200 Hz and necessitates digital filtering, the PowerTrack^TM^ delivers unprocessed output signals, maintaining fidelity with the raw biomechanical data. In terms of accuracy, the manufacturer has indicated an absolute ranging error of ±3% under calibration conditions and a typical resolution of 1 mm in high-precision configurations. Although it is based on ToF laser technology rather than LPT, the capacity of PowerTrack^TM^ to modulate measurement parameters could constitute a clear advantage for optimizing data acquisition in both experimental research and applied monitoring of eccentric resistance exercise.

The back squat protocol required participants to begin in an upright stance with knees and hips fully extended, feet positioned at approximately shoulder width, and the Smith machine barbell resting securely across the upper back and shoulders. From this position, subjects executed the eccentric action by descending until achieving 90° knee flexion, a target consistently standardized through the use of a mechanical stop to guarantee uniform depth across repetitions. Subsequently, the concentric phase was carried out at the highest intended velocity, provided that proper execution was preserved and safety of the participant was not jeopardized. The eccentric phase of the back squat performed on the Smith machine was executed in a controlled *ab libitum* manner once maximally intended velocity had been attained during the concentric phase. This methodological approach was deliberately adopted, as the purpose of the study was not to elicit improvements in eccentric performance, but rather to ensure ecologically valid and reproducible execution conditions that would allow for a valid and reliable evaluation of both measurement systems. In this context, the absence of a rigid temporal constraint during the descent avoided the imposition of artificial movement patterns that could differentially affect sensor outputs. The dependent variables analyzed were eccentric phase mean velocity (MV) and eccentric phase peak velocity (Vmax).

### 2.4. Statistical Analysis

All performance variables of the EP (MV and Vmax) followed a normal distribution as verified by the Kolmogorov–Smirnov test. The concurrent validity of the PowerTrack^TM^ in comparison with the criterion LPT—considered the gold standard—was examined through ordinary least products (OLP) regression [20]. To further evaluate agreement between systems, Lin’s concordance correlation coefficient (CCC) was calculated [21]. The interpretative thresholds for CCC were defined as “almost perfect” (>0.99), “substantial” (0.95–0.99), “moderate” (0.90–0.95), and “poor” (<0.90) [21]. In addition, Bland–Altman analyses were applied to determine systematic bias and the 95% limits of agreement between devices [22]. Reliability was established using the standard error of measurement (SEM), the intraclass correlation coefficient (ICC), and the coefficient of variation (CV), with values derived from inter-session variability across two independent testing days. Reliability was considered acceptable when ICC values exceeded 0.70 and the SEM remained below 0.20 [23]. The minimum detectable change (MDC) was computed as an index of sensitivity according to the formula (√2 × SEM × 1.96) [24]. All statistical analyses and graphical outputs were conducted with Python version 3.12 (Python Software Foundation, Beaverton, OR, USA).

## 3. Results

### 3.1. Validity

The concurrent validity analyses between the optoelectronic system (PowerTrack^TM^) and the criterion device (MuscleLab^TM^) demonstrated a very strong association for all eccentric phase velocity variables assessed during the Smith machine back squat.

**Figure 2:**
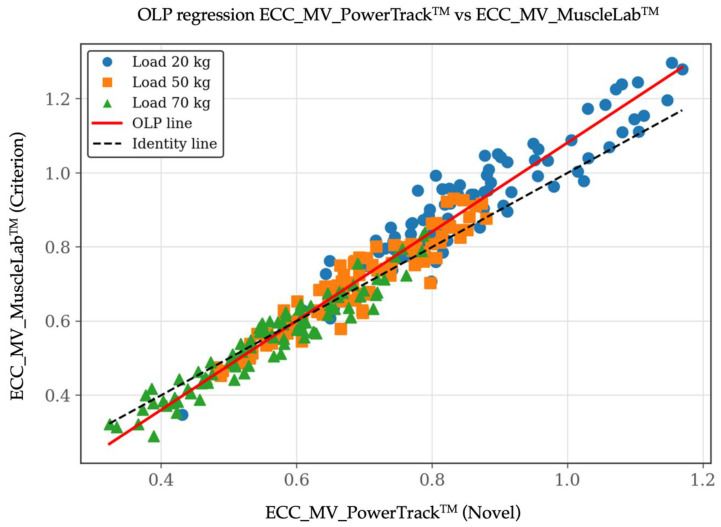
Validity test results by ordinary least products (OLP) regression plot between the PowerTrack^TM^ device (*X*-axis) and the MuscleLab^TM^ device (*Y*-axis) for eccentric mean velocity [MV at 20, 50, and 70 kg]. Data are shown in m/s.

**Figure 3:**
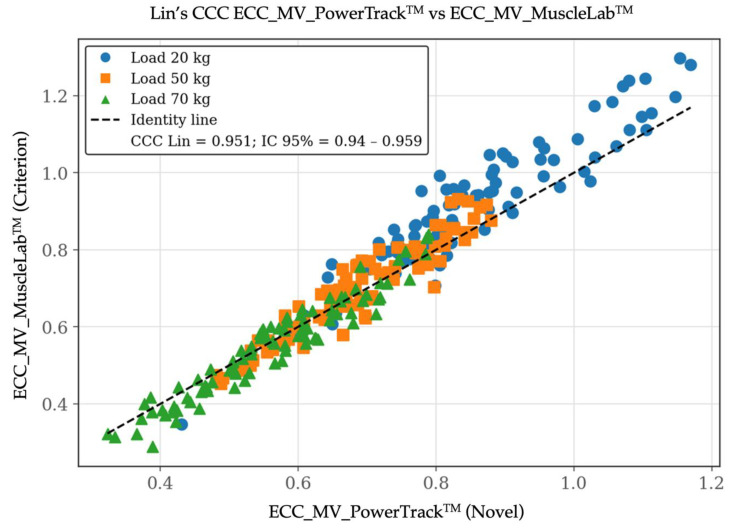
Validity test results by Lin’s concordance correlation coefficient (CCC) plot between the PowerTrack^TM^ device (*X*-axis) and the MuscleLab^TM^ device (*Y*-axis) for eccentric mean velocity [MV at 20, 50, and 70 kg]. Data are shown in m/s.

**Figure 4:**
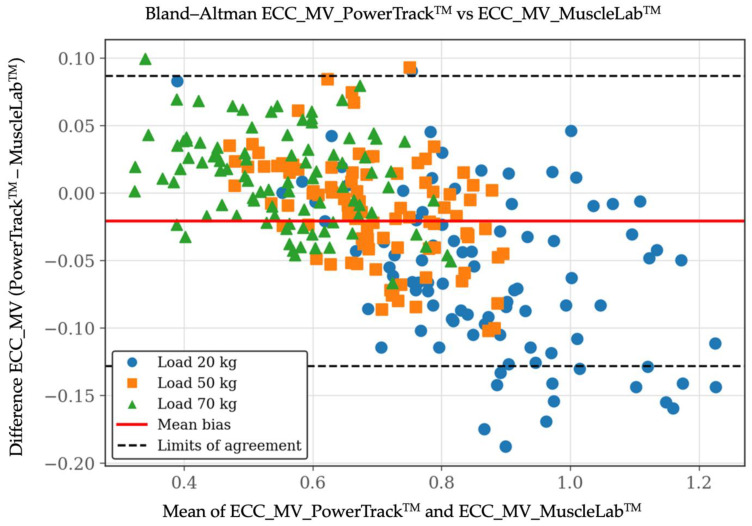
Validity test results by Bland–Altman plot between the PowerTrack^TM^ device (*X*-axis) and the MuscleLab^TM^ device (*Y*-axis) for eccentric mean velocity [MV at 20, 50, and 70 kg]. The Bland–Altman plot depicts the averaged difference and 95% limits of agreement (dashed lines). Data are shown in m/s.

For MV, ordinary least products (OLP) regression revealed a high correlation between methods (r = 0.97). However, both proportional and constant bias were identified. The OLP slope was 1.20 (95% CI: 1.17–1.24) and the intercept was −0.12 (95% CI: −0.14 to −0.10), indicating that PowerTrack^TM^ systematically underestimated MV relative to the criterion across the observed range. This pattern was particularly evident at higher eccentric velocities. Despite this bias, Lin’s concordance correlation coefficient (CCC) indicated very good agreement (CCC = 0.95; 95% CI: 0.94–0.96). Bland–Altman analysis for MV showed a small positive mean bias of 0.021 m·s^−1^, (95% CI: −0.087 to 0.128 m·s^−1^), suggesting consistent disagreement rather than random dispersion across measurements. These results revealed that bias magnitude and dispersion increased under length–load/higher eccentric velocity conditions, consistent with the proportional bias identified in the OLP regression. This behavior likely reflected the greater biomechanical and neuromuscular complexity of fast eccentric actions, particularly when using time-averaged metrics such as MV. Importantly, although systematic bias increased with velocity, overall agreement remained limited interchangeability rather than compromising the practical utility of the device for eccentric velocity monitoring. Figure 2, Figure 3 and Figure 4 include the validity test results for MV.

**Figure 5:**
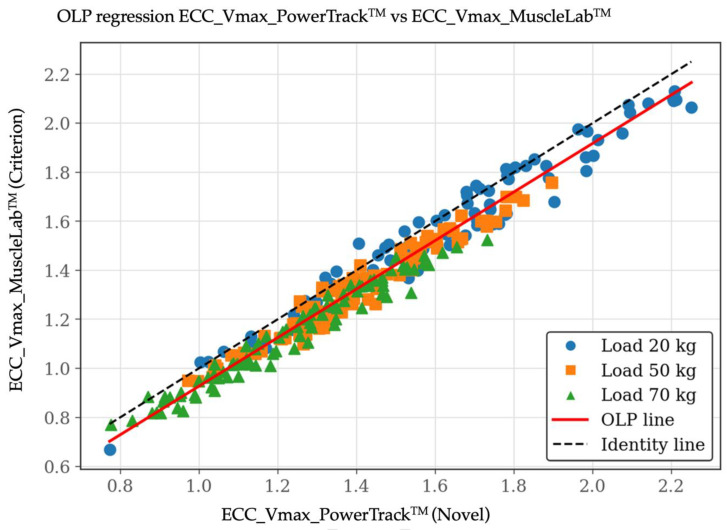
Validity test results by ordinary least products (OLP) regression plot between the PowerTrack^TM^ device (*X*-axis) and the MuscleLab^TM^ device (*Y*-axis) for eccentric peak velocity [Vmax at 20, 50, and 70 kg]. Data are shown in m/s.

**Figure 6:**
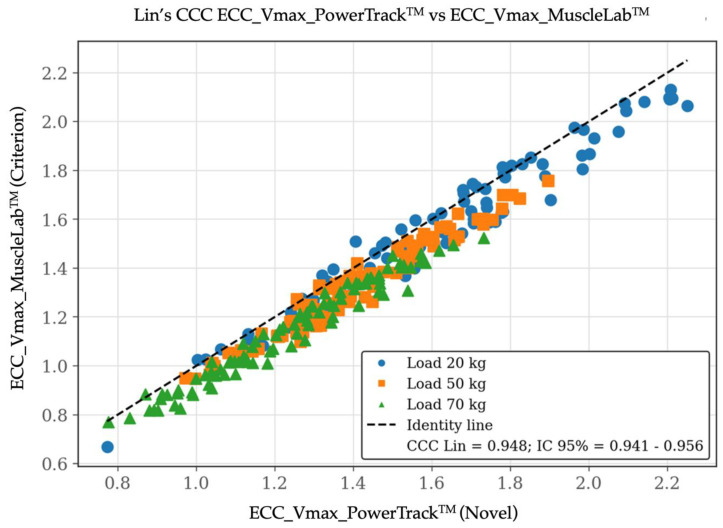
Validity test results by Lin’s concordance correlation coefficient (CCC) plot between the PowerTrack^TM^ device (*X*-axis) and the MuscleLab^TM^ device (*Y*-axis) for eccentric peak velocity [Vmax at 20, 50, and 70 kg]. Data are shown in m/s.

**Figure 7:**
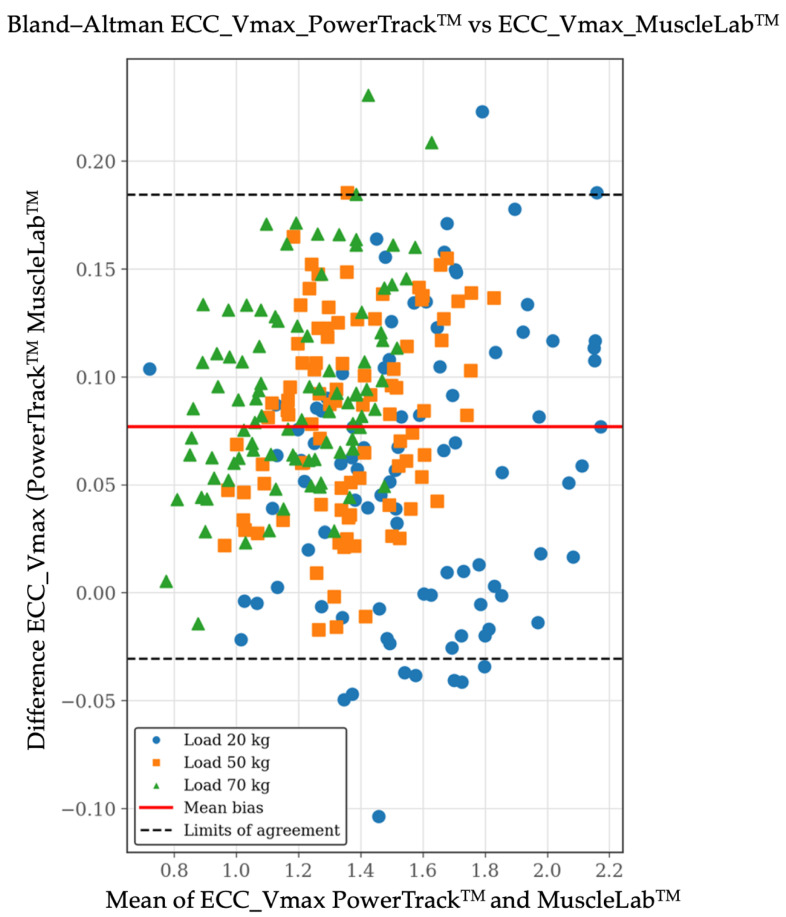
Validity test results by Bland–Altman plot between the PowerTrack^TM^ device (*X*-axis) and the MuscleLab^TM^ device (*Y*-axis) for eccentric maximum velocity [Vmax at 20, 50, and 70 kg]. The Bland–Altman plot depicts the averaged difference and 95% limits of agreement (dashed lines). Data are shown in m/s.

For Vmax, validity outcomes were more favorable. The correlation between systems was extremely high (r = 0.98), and the OLP regression slope was close to unity (0.99: 95% CI: 0.97–1.01), indicating no proportional bias. Nevertheless, a small but significant constant bias was observed, as reflected by a negative intercept (−0.06; 95% CI: −0.09 to −0.03). Lin’s CCC confirmed a very high level of concordance (CCC = 0.95; 95% CI: 0.94–0.96), with minor reductions relative to Pearson’s r, attributable primarily to systematic bias rather than increased variability. The Bland–Altman plot for Vmax revealed a mean bias of −0.077 m·s^−1^ (95% CI: −0.184 and 0.031 m·s^−1^), indicating a small and consistent offset between devices. Figure 5, Figure 6 and Figure 7 include the validity test results for Vmax.

### 3.2. Reliability

Agreement analysis using intraclass correlation coefficients demonstrated excellent consistency between systems for eccentric phase velocity metrics. The novel device (PowerTrack^TM^) exhibited excellent relative reliability (ICC range, 0.87–0.96) for each variable assessed regardless of the load tested (Table 1). Additionally, the CV and SEM were lower than 8% and 0.04 for each load, respectively, indicating the high precision of the assessments.

## 4. Discussion

The present study provides a comprehensive evaluation of the concurrent validity and reliability of an optoelectronic system for assessing eccentric phase velocity during a multi-joint resistance exercises. The findings contribute novel methodological evidence to a field in which the physiological relevance of eccentric actions is well established, yet the objective quantification of eccentric velocity remains insufficiently developed.

### 4.1. Eccentric Velocity as a Physiologically Relevant but Methodologically Undeveloped Variable

Eccentric muscle actions are characterized by distinct neuromuscular and mechanical properties compared with concentric contractions, including higher force capacity at lower metabolic cost, altered motor unit behavior, and unique muscle–tendon interactions [25,26]. These characteristics underpin the widespread interest in eccentric training for both performance enhancement and clinical applications [2].

Several experimental studies have demonstrated that the velocity of eccentric actions plays a critical role in modulating muscle hypertrophy, strength adaptations, and fiber-type-specific responses [3,4]. More recently, Amstrongt et al. [27] showed that eccentric force–velocity profiles differed substantially from concentric profiles and exhibited pronounced inter-individual variability, reinforcing the notion that eccentric performance cannot be inferred from concentric assessment.

Recent evidence indicates that eccentric training may produce slightly greater strength gains, while eliciting comparable improvements in hypertrophy, power, and functional capacity in older adults compared to conventional training, thereby representing a promising intervention for the management of sarcopenia and dynapenia [28].

Despite this body of physiological evidence, the instrumental validation of eccentric velocity has lagged. As highlighted in the narrative review published by Handford et al. [29], most resistance training studies had either uncontrolled eccentric phases or relied on indirect timing strategies (e.g., tempo prescriptions), rather than objective velocity measurements. To the best of our knowledge, few studies have systematically validated eccentric phase velocity metrics—such as MV or Vmax—using formal agreement analyses against a criterion system in multi-joint exercises. The present study therefore addresses a genuine methodological gap by aligning measurement technology with contemporary understanding of eccentric muscle function.

### 4.2. Interpretation of High Agreement in the Presence of Systematic Bias

The high concordance observed between the optoelectronic device and the criterion system (CCC and ICC values approximately 0.95) indicates that the eccentric velocity can be quantified with a level of agreement comparable to that reported for concentric velocity in prior validation studies [17,20,30]. This finding is particularly noteworthy given the complexity and variability inherent to eccentric actions.

The detection of proportional and constant bias, especially for eccentric mean velocity, should not be interpreted as a failure of the measurement system. Instead, it reflects the intrinsic characteristics of eccentric movement. Armstrong et al. [27] demonstrated that even under isovelocity conditions, eccentric force production showed substantial variability due to neural inhibition, task-dependent control strategies, and muscle–tendon dynamics. When eccentric actions are performed freely, as in the present study, such variability is expected to influence time-averaged metrics more strongly than instantaneous or peak measures.

From an applied validity perspective, these results are consistent with recommendations that emphasize agreement and consistency over perfect interchangeability when validating tools for field-based use [22,31]. The systematic bias observed in this study delineates the operational limits of the device, but it does not preclude its use for monitoring changes over time or for prescribing eccentric training within a consistent measurement framework.

### 4.3. Differential Robustness of Eccentric Velocity Metrics: Mean Velocity Versus Maximal Velocity

One of the most relevant findings of the present study is the superior validity profile of Vmax compared with MV. This distinction is supported by both biomechanical reasoning and empirical evidence. Eccentric movements are inherently non-uniform, involving continuous modulation of braking forces, anticipatory motor control, and stiffness regulation throughout the descent phase [26,32].

Metrics based on averaging over time, such as MV, are therefore more susceptible to fluctuations in descent strategy, depth-specific deceleration, and inter-repetition variability [15,27]. In contrast, Vmax reflects a discrete event within the eccentric phase that may be less affected by these factors. Previous studies examining eccentric tempo and duration have reported substantial variability in average velocity measures while showing more stable peak values [15,29].

These findings have important methodological implications. They suggest that not all velocity-derived variables are equally appropriate for eccentric monitoring, and that peak-based metrics may offer greater robustness and sensitivity for applied eccentric velocity-based training. This consideration is particularly relevant when developing monitoring frameworks for populations with heterogeneous movement strategies, such as clinical cohorts.

### 4.4. Translational Implications for Eccentric Training Prescription

The validation of eccentric velocity monitoring in a widely used multi-joint exercise such as the Smith machine back squat has direct practical relevance. Eccentric training has been increasingly promoted in health-related contexts due to its favorable force-to-metabolic-cost ratio and its capacity to induce neuromuscular adaptations at relatively low perceived exertion [2]. Nevertheless, the absence of objective tools to dose and monitor eccentric loading has limited its broader adoption.

Beyond health applications, the ability to monitor eccentric velocity also holds substantial implications for athletic performance. Many sports (e.g., football, basketball, handball) are characterized by repetitive or abrupt eccentric actions, such as deceleration, landing, cutting, and change-of-direction tasks, where eccentric force production and braking capacity are critical determinants of performance and injury risk. In these contexts, inadequate eccentric control has been associated with both reduced performance efficiency and a higher prevalence of musculotendinous injuries, particularly in high-demand muscle groups such as the knee extensors and hamstrings [1,33].

By demonstrating that eccentric velocity—particularly Vmax—can be measured with high reliability and agreement, the present study supports the feasibility of eccentric velocity-based training as a practical framework for both performance enhancement and injury risk management. Objective monitoring of eccentric velocity may allow practitioners to better regulate braking demands, identify excessive or insufficient eccentric loading, and individualize training stimuli based on the athlete’s mechanical capacity rather than relying solely on external load prescriptions.

This approach aligns with contemporary training paradigms that emphasize individualized mechanical loading and movement-specific constraints over fixed percentages of one-repetition maximum, especially in scenarios where fatigue accumulation and joint stress must be carefully managed [1]. Importantly, the ability to quantify eccentric velocity in multi-joint exercises provides a bridge between laboratory-derived knowledge on eccentric mechanics and real-world training environments, facilitating the integration of eccentric-focused strategies within comprehensive performance and injury–prevention programs.

### 4.5. Limitations and Future Directions

Despite these translational implications, several limitations of the present study should be acknowledged when interpreting the findings. First, the validation has been conducted using a single exercise modality—the back squat performed on a Smith machine—which provides a controlled movement trajectory but may limit the generalizability of the results to free-weight exercises or movements involving greater degrees of freedom. Although the Smith machine offers advantages for standardization and reproducibility, eccentric velocity profiles may differ in exercises that require higher levels of postural control and intermuscular coordination.

Second, the loading conditions examined were restricted to a specific range of absolute loads. While this approach allows for consistent comparisons between measurement systems, it does not capture the full spectrum of eccentric velocity behaviors that may occur under heavier loads, near-maximal intensities, or during ballistic or stretch-shortening cycle-dominant tasks. Consequently, caution is warranted when extrapolating the present findings to training scenarios emphasizing maximal eccentric overload or highly explosive movements.

Third, the eccentric phase was performed in a controlled *ab libitum* manner without an externally imposed tempo. Although this decision is methodologically justified to ensure ecological validity and to avoid artificially constraining movement strategies, it may have contributed to increased inter-repetition variability, particularly for time-averaged metric such as MV. Future studies could systematically compare controlled tempo versus self-selected eccentric executions to further elucidate how the execution strategy influences measurement agreement.

Additionally, the present investigation focused exclusively on kinematic variables (MV and Vmax) and does not examine their relationship with kinetic or physiological outcomes, such as force production, muscle activation patterns, or marker of neuromuscular fatigue. As a result, the functional and biological significance of small systematic biases in eccentric velocity remains to be fully established.

Future research should aim to extend the validation of eccentric velocity monitoring to a broader range of exercises, including free-weight and sport-specific movements, as well as to different populations, such as trained athletes, older adults, and clinical cohorts. Of particular interest is the application of this monitoring approach to elastic resistance exercise and its potential association with rates of perceived exertion in older adults.

Longitudinal studies are also needed to determine the sensitivity of eccentric velocity metrics to training-induced adaptations and their utility for guiding load progression, fatigue management, and injury risk mitigation. Finally, integrating eccentric velocity measurements with kinetic, electromyographic, and performance-based outcomes would provide a more comprehensive framework for understanding the role of eccentric velocity in both health- and performance-oriented training paradigms.

## 5. Conclusions

This study demonstrated that eccentric phase velocity during the Smith machine back squat can be assessed with high concurrent validity and reliability using an optoelectronic system. Both MV and Vmax showed very high agreement with the criterion device, although small and consistent systematic biases were observed. Notably, eccentric peak velocity exhibited a more robust validity profile than MV, suggesting greater suitability for applied monitoring. These findings indicated that eccentric velocity can be quantified reliably under ecologically valid training conditions. The validation of this approach could support the feasibility of eccentric velocity-based training in both health and performance contexts. Overall, the present results can contribute to closing an important methodological gap by providing objective support for the practical monitoring and prescription of eccentric training.

## Figures and Tables

**Table 1:** Reliability of eccentric mean velocity (MV) and eccentric peak velocity (Vmax) obtained from PowerTrack^TM^ at different loads during the Smith machine back squat exercise.

Device	Variable	Load (kg)	CV (%)	MDC	ICC (95% CI)	SEM
PowerTrack^TM^	MV	20	6.86	0.058	0.87 (0.79–0.95)	0.021
		50	5.74	0.029	0.92 (0.89–0.95)	0.010
		70	6.30	0.020	0.95 (0.93–0.97)	0.007
	Vmax	20	7.73	0.087	0.90 (0.86–0.94)	0.030
		50	6.62	0.058	0.93 (0.90–0.96)	0.018
		70	6.01	0.053	0.96 (0.94–0.98)	0.011

CV: coefficient of variation; MDC: minimum detectable change; ICC: intraclass correlation coefficient; CI: confidence interval; SEM: standard error of measurement; MV: mean velocity; Vmax: peak velocity.

## Data Availability

Data are available from the corresponding author upon reasonable request. The dataset was also uploaded to the journal submission platform during the manuscript submission process.

## Abbreviations

The following abbreviations are used in this manuscript:

1RM	One-Repetition Maximum
CCC	Lin’s Concordance Correlation Coefficient
CI	Confidence Interval
CV	Coefficient of Variation
ICC	Intraclass Correlation Coefficient
LPT	Linear Position Transducer
MDC	Minimum Detectable Change
MV	Mean Velocity
OLP	Ordinary Least Products Regression
SEM	Standard Error of Measurement
ToF	Time-of-Flight
VBT	Velocity-Based Training
Vmax	Maximum Velocity
